# Segmental Orthodontics for the Correction of Cross Bites

**DOI:** 10.5005/jp-journals-10005-1080

**Published:** 2011-04-15

**Authors:** Anirudh Agarwal, Rinku Mathur

**Affiliations:** 1 Professor and Head, Department of Orthodontics, Rajasthan Dental College and Hospital Jaipur, Rajasthan, India; 2 Assistant Professor, Department of Pedodontics and Preventive Dentistry, Government Dental College and Hospital Jaipur, Rajasthan, India

**Keywords:** Anterior and posterior cross bite, 2 × 4 appliance, Lingual button with cross elastics.

## Abstract

Cross bite is a condition where one or more teeth may be abnormally malposed buccally or lingually or labially with reference to the opposing tooth or teeth. Cross bite correction is highly recommended as this kind of malocclusion do not diminish with age. Uncorrected cross bite may lead to abnormal wear of lower anteriors and cuspal interference, mandibular shift resulting in mandibular asymmetry and temporomandibular joint dysfunction syndrome. There are several methods for treating this type of malocclusion. In this article, segmental orthodontics has been highlighted by using 2 × 4 appliance therapy and lingual button with cross elastics. This appliance offers many advantages as it provides complete control of anterior tooth position, is extremely well tolerated, requires no adjustment by the patient and allows accurate and rapid positioning of teeth.

## INTRODUCTION

Cross bite is a condition where one or more teeth may be abnormally malposed buccally or lingually or labially with reference to the opposing tooth or teeth. Based on location they can be anterior and posterior. Which can be further classified on the basis of nature as skeletal, dental and functional cross bites.

The term anterior cross bite is used to describe an abnormality in the anterior/posterior plane, whereby a mandibular tooth is positioned anterior to a maxillary tooth. Posterior cross bite refers to an abnormal transverse relationship between the maxillary and mandibular teeth.

If the condition arises only from palatal malposition of a maxillary tooth with associated labioversion of contacting mandibular teeth, it may be called ‘dental’ cross bite.^1^ A patient with a prognathic mandible may also have anterior cross bite even though the teeth are positioned normally over the maxillary and mandibular basal bone. Such malocclusion is termed ‘skeletal’ anterior cross bite and is associated with class III malocclusion. Skeletal posterior cross bites are usually characterized by a narrow maxillary arch.

Dental anterior cross bite, has a reported incidence of four to five percent, usually becomes evident during the early mixed dentition phase^2-5^ and is generally the result of an abnormal eruption of the permanent incisors. A variety of etiological factors may be involved including trauma to the primary incisor with displacement of the permanent tooth bud; delayed exfoliation of the primary incisors with palatal deflection of the erupting permanent incisor; supernumerary anterior teeth; odontomas or crowding in the incisor region.^2-7^

Posterior cross bite is a common malocclusion of the primary and mixed dentition. An insufficient maxillary arch width, typically results in a unilateral posterior cross bite with an associated lateral mandibular displacement (also known as mandibular deviation or shift). When the maxilla is severely constricted, a bilateral posterior cross bite develops.

Different techniques have been used to correct dental cross bite. As the affected patients are young and ideally any appliances needed for correction of dental cross bite should be easily placed and removed, inexpensive, comfortable and easily tolerated, should not damage the associated tissue, give rapid cross bite correction and require only minimal patient cooperation.

This article presents the cases related to, management of anterior and posterior cross bite using fixed segmental orthodontic therapy for mixed and permanent dentition.

## CASE 1

**Fig. 1:**
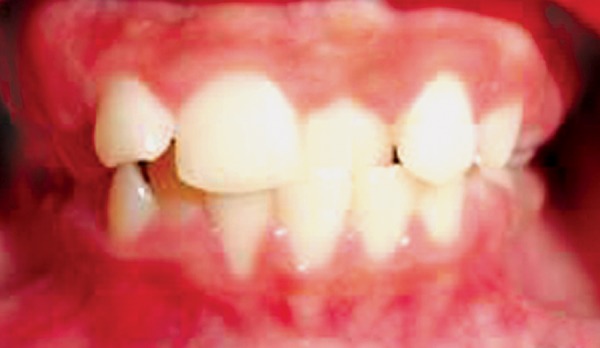
Preoperative showing 21 in cross bite

A 11-year-old boy was referred for the treatment of anterior cross bite. On examination, he presented with class I molar and canine relationship. Cross bite was seen in relation to 21 and lower anterior segment had mild crowding (Fig. 1). Arch form was ovoid, with no rotations.

**Fig. 2:**
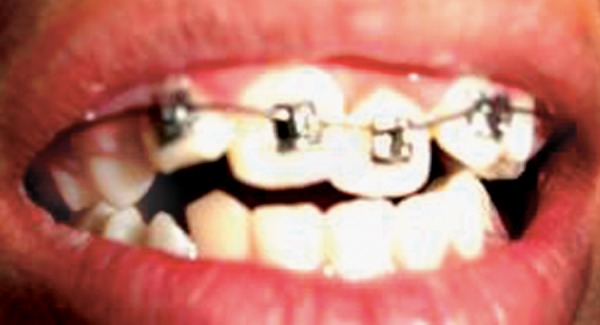
2 × 4 appliance in place

**Fig. 3:**
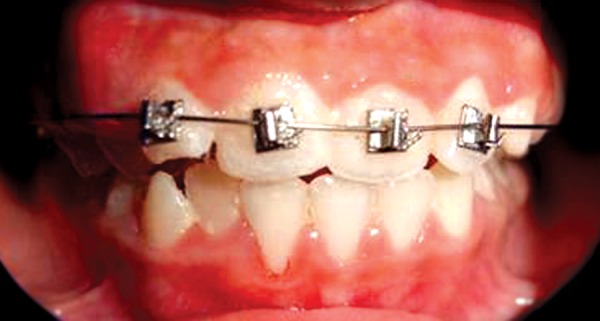
Corrected cross bite after 14 days

**Fig. 4:**
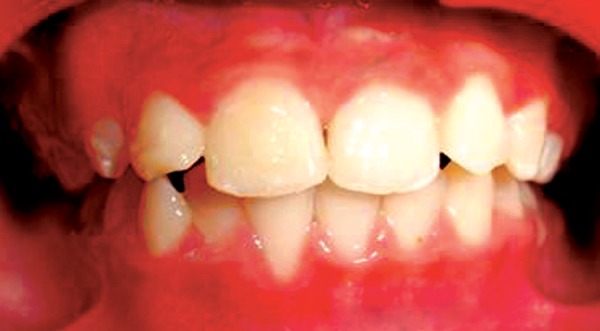
After debonding

**Fig. 5:**
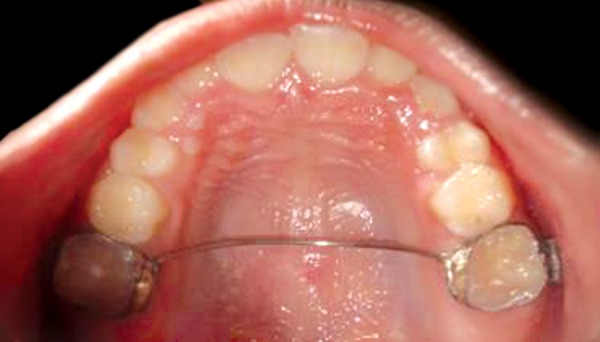
Transpalatal arch

After occlusal assessment oral prophylaxis was done and APF gel was applied. The treatment plan comprised of 2 × 4 appliance therapy. Maxillary incisors were bonded with Begg’s brackets. NiTi wire 0.014 inch with ovoid arch form engaged (Fig. 2). Occlusal clearance for 21 was done with composite build up on deciduous molars. The patient was advised soft diet and recalled after one week to evaluate the progress. Rapid correction of the incisor relationship occurred and the patient was debonded after two weeks of bonding (Figs 3 to 5) and temporary composite build ups were removed. Correction of the cross bite was rapid. No retainers were considered necessary as the overbite prevented the risk of relapse. The patient was kept under review with occlusion remaining stable even one year later.

## CASE 2

A 14-year-old boy was referred for orthodontic management of his upper right incisors, which had erupted in cross bite. He presented with bilateral class I molar relation with permanent dentition. Treatment of cross bite was accomplished in two phases.

In phase I, maxillary expansion was planned to provide room for labial tipping of 11, 12 with removable appliance (Fig. 7).

Postinsertion instructions were given and patient was advised soft diet. Seven days after wearing of appliance, the jack screw was activated. The activation method was demonstrated and instructed to the patient. Patient was reviewed every fortnight. Two months later enough room was achieved for 11, 12.

In phase II and fixed segmental orthodontic therapy with Begg’s brackets for labial tipping of 11, 12, was planned. Begg’s brackets were bonded from 14 to 24. NiTi wire of 0.014 inch with ovoid arch form was engaged (Fig. 8). Additional space was gained through mesial stripping of 13 followed with fluoride varnish application.

The jack screw was sealed with self-cure and was used as a retainer appliance and in order to obtain the occlusal clearance. At each fortnight, the patient was reviewed and checked for the correction and oral hygiene status. After four weeks, when 11 and 12 were unlocked, the posterior bite plane was trimmed off.

**Fig. 6:**
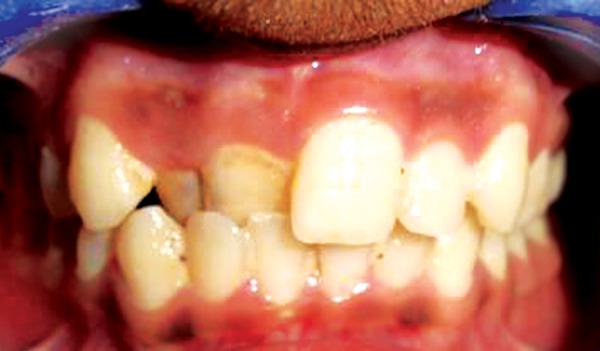
Preoperative showing 11, 12 in cross bite

**Fig. 7:**
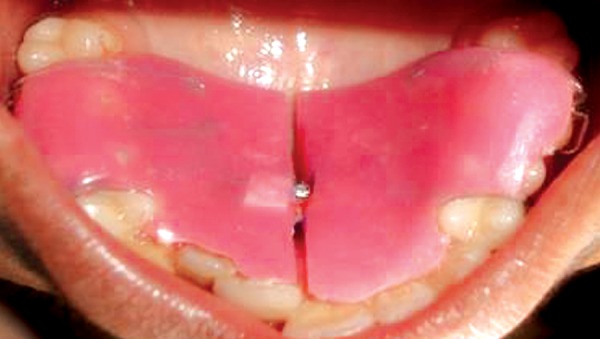
Removable jack screw appliance with posterior bite planes

**Fig. 8:**
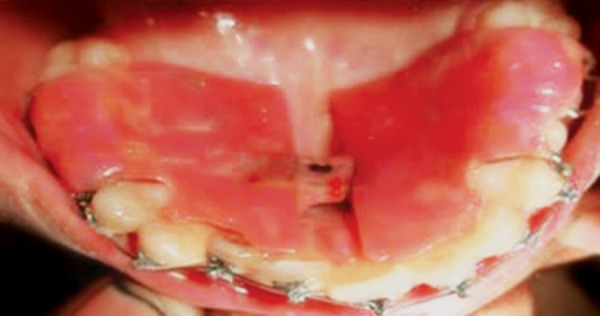
Begg’s brackets from 14 to 24

**Fig. 9:**
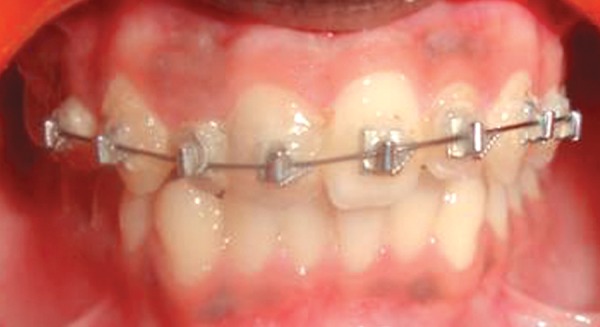
Correction of cross bite

**Fig. 10:**
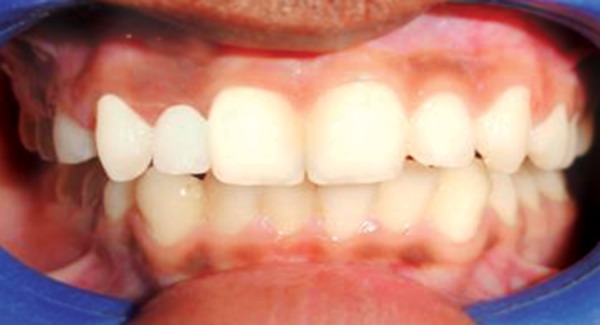
After debonding

**Fig. 11:**
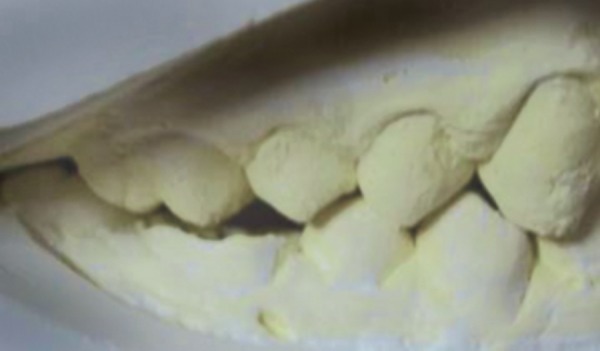
Preoperative showing posterior cross bite in relation to 16 and 46

Rapid correction of the incisor relationship occurred. Debonding was done three weeks later, when the anterior-posterior arch relationship with respect to 11and 12 were corrected (Figs 9 and 10). Hawleys appliance was delivered for retention. At 6-month review this retainer had been lost but the positive overbite had maintained the overjet correction.

## CASE 3

**Fig. 12:**
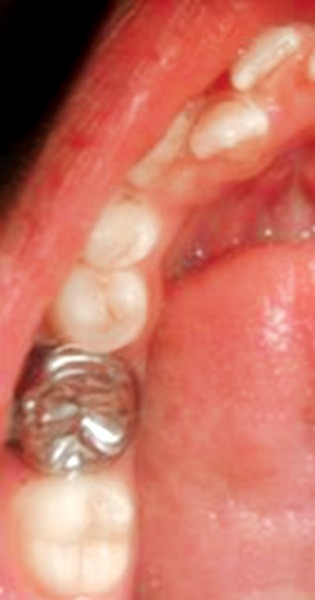
Stainless steel crown with welded buccal tube

A 14-year-old boy was referred for difficulty in chewing from right side. He presented with grossly decayed 46 which was also in cross bite with 16 (Fig. 11). The molar relation on left was class I. Orthodontic correction of the cross bite was preceded by endodontic therapy of 46. Stainless steel crown with welded buccal tube was cemented on 46 (Fig. 12). Interarch elastic method, which is based on reciprocal intermaxillary force principle was opted for correction of this individual tooth cross bite.

**Fig. 13:**
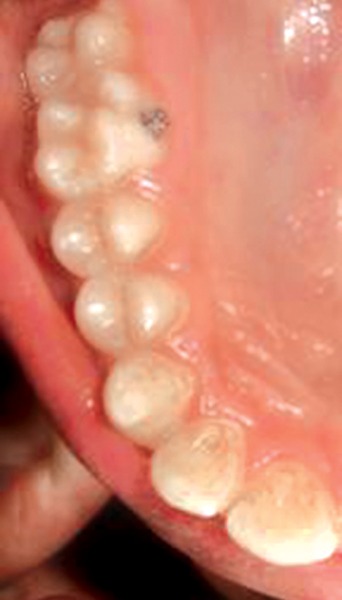
Lingual button on palatal of 16

**Fig. 14:**
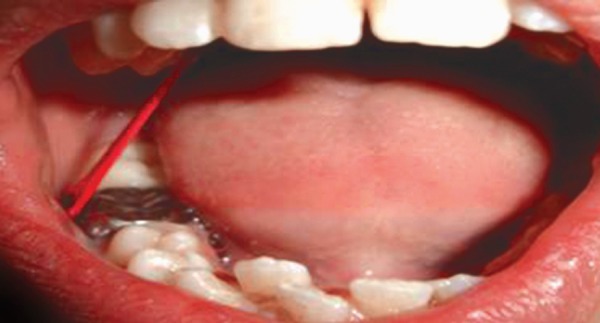
Interarch elastics engaging buccal

**Fig. 15:**
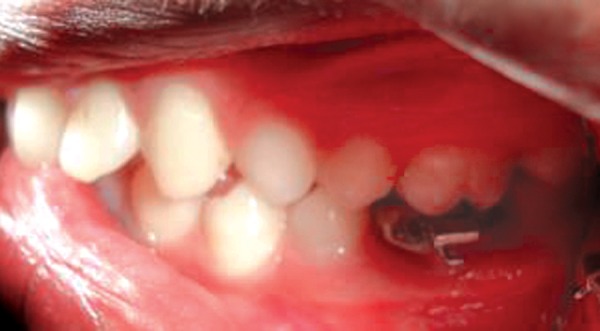
Postoperative showing corrected 46

**Fig. 16:**
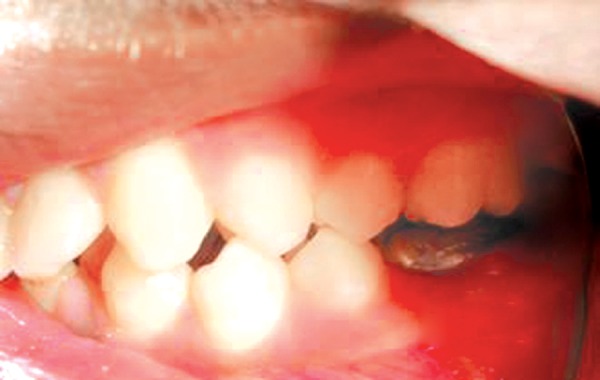
After debonding

A lingual button was bonded on the palatal surface of 16 (Fig. 13) and interarch elastic was engaged between lingual of 16 and buccal of 46 (Fig. 14). The patient was instructed to wear elastics for full time and were to be replaced once in 24 hours. After 8 weeks of the therapy, cross bite correction was achieved with established Angle’s class I molar relation (Figs 15 and 16).

## DISCUSSION

Cross bites if left untreated can lead to serious oral health problems like a traumatic occlusion can occur, resulting in attrition of teeth, mobility and apical migration of labial gingivae. A functional cross bite can also develop from cuspal interference resulting in a mandibular shift. This may lead to an apparent mandibular asymmetry and may cause temporomandibular joint dysfunction syndrome. Hence, early treatment is important to re-establish proper muscle balance by elimination of occlusal interference.^8^

In case 1 and 2, the 2 × 4 appliance therapy has been used which is advantageous over the conventional removable appliances. In this appliance continuous arch wires are used which provide complete control of the anterior dentition as well as maintain a good arch form. Rapid correction of malocclusion takes place in a single short phase of therapy. 2 × 4 offers a more effective and efficient tooth positioning and allows three-dimensional control of the involved teeth during correction of anterior cross bite. This only requires more chairside time to fit the appliance and there is no laboratory cost involved. The major factor which determined the success of anterior cross bite correction was achievement of positive overbite. If a positive overbite can be established then the prognosis for maintaining the corrected cross bite is good.

In cases where there is a major discrepancy in the inclination of the upper incisors, it may be necessary to use fixed appliance along with a rectangular nickel-titanium wire or even something more rigid, such as a TMA wire to correct the inclinations ofthe incisor teeth. Although patient cooperation is necessary during placement, maintenance and removal of the fixed appliance is less than that required with a removable appliance. The other disadvantages of removable appliances are that they are rarely worn fulltime, there is difficulty in speech, eating, gagging, decalcification, caries, gingivitis, palatal, hyperplasia, fungal infections, incorrect activation produces unhelpful changes and allow only tipping of teeth, etc.^9^

It is, of course, essential that the patient is able to maintain an adequate standard of oral hygiene, in view of the increased risks of decalcification and caries associated with fixed appliances.

The most appropriate timing of treatment occurs, when the patient is in the late deciduous or early mixed dentition stage.^10^ As expansion modalities are very successful in this age group and permanent incisors are given more space as a result of the expansion. However, in case 2, we have used a combination of removable expansion appliance and segmental fixed orthodontic therapy, which has also given good results.

The treatment of posterior dental cross bite has been regarded as the correction of the buccolingual tipping of a single or group of teeth and re-establishing the balance between the upper and lower arches. The use of interarch elastics in the treatment of single tooth posterior cross bites provided satisfactory results restoring the functional contact with the antagonist teeth. Lingual buttons are less bulky and more esthetic, no laboratory procedures are required, bonding is quick and easy, and the fixed appliance therapy can be carried out simultaneously.

## CONCLUSION

The cases presented here, were treated with 2 × 4 and cross elastics, all the cases gave satisfactory and stable results in short duration.
